# The Endovascular Treatment of Epistaxis

**DOI:** 10.5334/jbr-btr.954

**Published:** 2015-12-30

**Authors:** S. Nicolay, T. Van Der Zijden, M. Voormolen, O. d’Archambeau, J. Maes, F. De Belder, P. M. Parizel

**Affiliations:** 1 Department of Radiology, Antwerp University Hospital (UZA) & University of Antwerp (UA), Belgium

**Keywords:** epistaxis, endovascular, angiography

## Abstract

Epistaxis or nosebleed is relatively common in the general population. Depending on the location of the bleeding in the nasal cavity, epistaxis can be divided in two types: anterior or posterior type. The anterior type is far more frequent, often self-limiting and, if needed, is relatively easy treatable. Posterior type epistaxis is rare and more likely to require medical attention. The cornerstone of the conservative therapy of posterior epistaxis is nasal packing. Only in patients with persistent or recurrent epistaxis, endovascular intervention or surgery is indicated. Both treatment options have a similar success and complication rate, but endovascular treatment, if feasible, has several advantages above surgical treatment. The choice of procedure should be made on a patient-to-patient basis, taking several parameters into account.

In this pictorial essay we present an overview of the relevant radiological anatomy and a review of various causes of epistaxis, with the emphasis on the endovascular treatment.

## Introduction

Epistaxis or nosebleed is relatively common in the general population. About 60% of people will experience one or more episodes of epistaxis in their lifetime, men and women equally affected. The aetiology can be divided into local and systemic. Local nasal causes include infection, inflammation, trauma, neoplasms, foreign bodies, irritative inhalants and iatrogenic. Systemic causes include pathologic or induced factors with increased risk of bleeding, such as the use of antiaggregant medication, systemic vascular disorders and to a lesser extent blood dyscrasias and haematological neoplasms [1]. Depending on the location of bleeding in the nasal cavity a distinction between anterior and posterior epistaxis can be made.

The majority of nose bleedings are self-limiting and originate in the anterior nasal cavity, more specific in the anterior septal area, vascularized by the Kiesselbach plexus, known as Little’s area. When anterior epistaxis is not responsive to simple measures like compression, medical treatment is necessary. This includes general measures like blood pressure control, fluid resuscitation and pain relief. Because the bleeding area is often clearly visible and readily accessible, anterior nasal packing, cautery or topical vasoconstriction will usually be successful [2].

About 5% of epistaxis episodes occur posterior in the nasal cavity. Treatment of posterior epistaxis is more challenging and initially, next to general measures, consists of both anterior and posterior nasal packing achieving adequate local compression and preventing nasopharyngeal aspiration as well. To prevent complications like infection, vasovagal reaction, hypoxia, myocardial infarction or even death, nasal packing should be removed within 48 hours [3].

In case of persistent or recurrent epistaxis despite optimal conservative treatment, endovascular treatment (embolization) or endoscopic surgery is indicated. The use of endovascular embolization as an alternative to surgery was introduced in the mid-seventies [4]. The overall success rate of endovascular treatment in terms of adequately stopping the bleeding (71–100%) is comparable to surgery [5]. The complication and failure rates were found to be similar as well. Embolization, however, is associated with more severe complications such as blindness and stroke, which can occur in case of significant anastomosis between the vascular territory of the nose and the eye or the brain. On the other hand, the endovascular approach is a cheaper and usually a less invasive alternative to surgery, mostly requiring no general anaesthesia. In daily practice, treatment modality choice should be done on a case-to-case basis, taking into account the patients comorbidities, vascular anatomy and availability of interventional radiology.

## Relevant vascular anatomy of the nose

**Figure 1:**
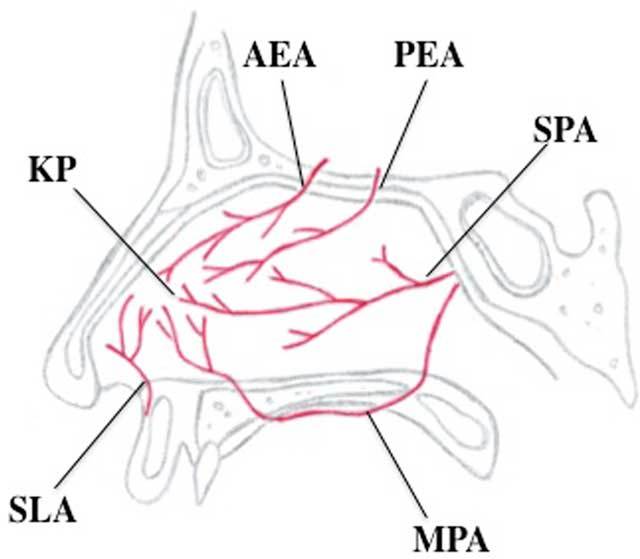
Illustration of the arterial supply of the nose. The anterior part of the nasal cavity is vascularized by the superior labial artery (SLA), a small vessel branching off the facial artery, which is a large side-branch of the external carotid artery. The anterior ethmoid artery (AEA) and posterior ethmoid artery (PEA) pass through the cribriform plate of the ethmoid, supplying the roof of the nose. Both vessels originate from the ophthalmic artery, which is the first major side-branch of the internal carotid artery. The posterior part of the nasal cavity, including most of the nasal septum and the lateral walls, is supplied by the sphenopalatine artery (SPA), which is the terminal branch of the maxillary artery, coming from the external carotid artery. The floor of the nose, the palate, is vascularized by the major palatine artery (MPA), branching off the distal maxillary artery. KP = Kiesselbach plexus [6,7].

**Figure 2:**
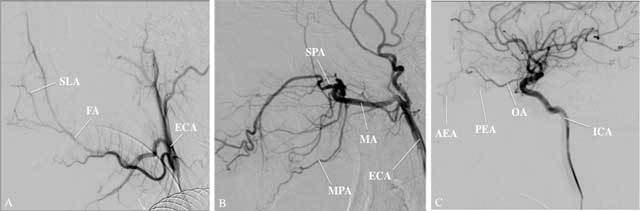
A: Lateral view angiogram in the arterial phase of the ECA showing its proximal segment with the SLA, branching off the facial artery (FA). B: Lateral view angiogram, arterial phase, of the distal segment of the ECA showing the SPA as the terminal branch of the maxillary artery (MA). The MPA is another branch of the distal MA, running caudally to the palate. C: Lateral view angiogram, arterial phase, of the ICA, showing the anterior ethmoid artery (AEA) and posterior ethmoid artery (PEA) originating from branches of the ophthalmic artery (OA).

**Figure 3:**
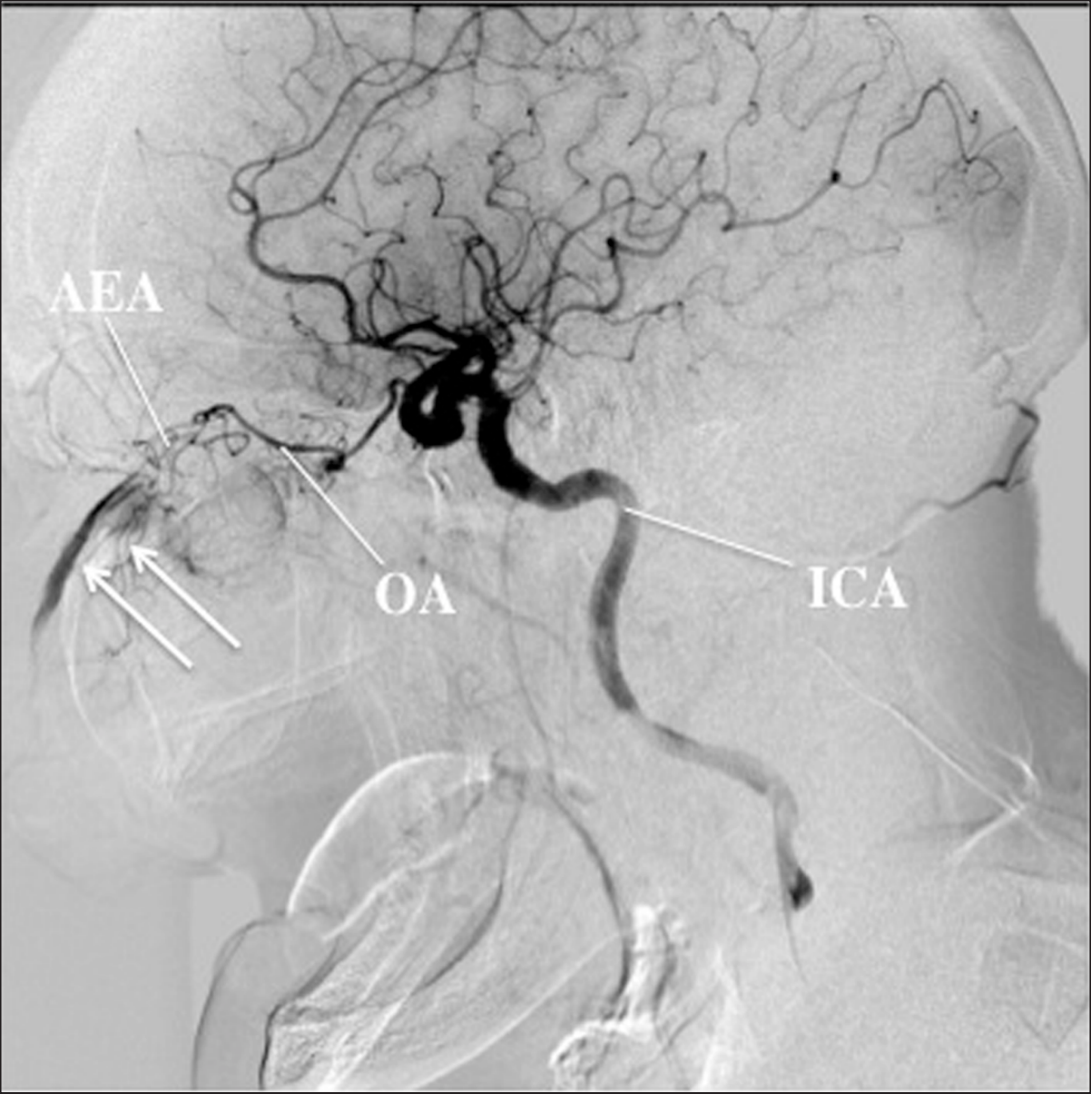
Angiography, lateral view, arterial phase, of the right ICA of a 43-year-old patient shows a large extravasation (arrows) in the anterior part of the nasal cavity, coming from the AEA in connection with the OA. In this case, no endovascular embolization was undertaken because of the risk of blindness. The patient was subsequently treated with surgical arterial ligation.

Several arteries provide blood to the mucosa of the nasal cavity (Fig. 1). Complete diagnostic angiograms of the internal carotid artery (ICA) and external carotid artery (ECA) are essential in the evaluation of epistaxis since both territories participate in vascularization of the nose [6] (Fig. 2A–C). The dominant supplying artery to the nasal cavity is the terminal branch of the maxillary artery (MA), the sphenopalatine artery (SPA). Branches of this vessel are involved in the majority of cases of refractory epistaxis, so this will be the primary target in most endovascular interventions. The floor of the nasal cavity is mainly vascularized by the major palatine artery (MPA), which is the main continuation of the descending palatine artery, also a vessel arising from the maxillary artery. The ethmoid arteries (EA), supplying the nasal roof, branch off the ophthalmic artery (OA). A nosebleed in the territory of the EA is a relative contraindication for embolization because of the risk of blindness (Fig. 3). The small superior labial artery (SLA) vascularizes part of the anterior nasal cavity.

The well-known Kiesselbach plexus, located in Little’s area in the anteroinferior part of the nasal septum, is a region where different nasal arteries anastomose [7]. Ninety percent of nosebleeds occur in this area and are often self-limiting.

**Figure 4:**
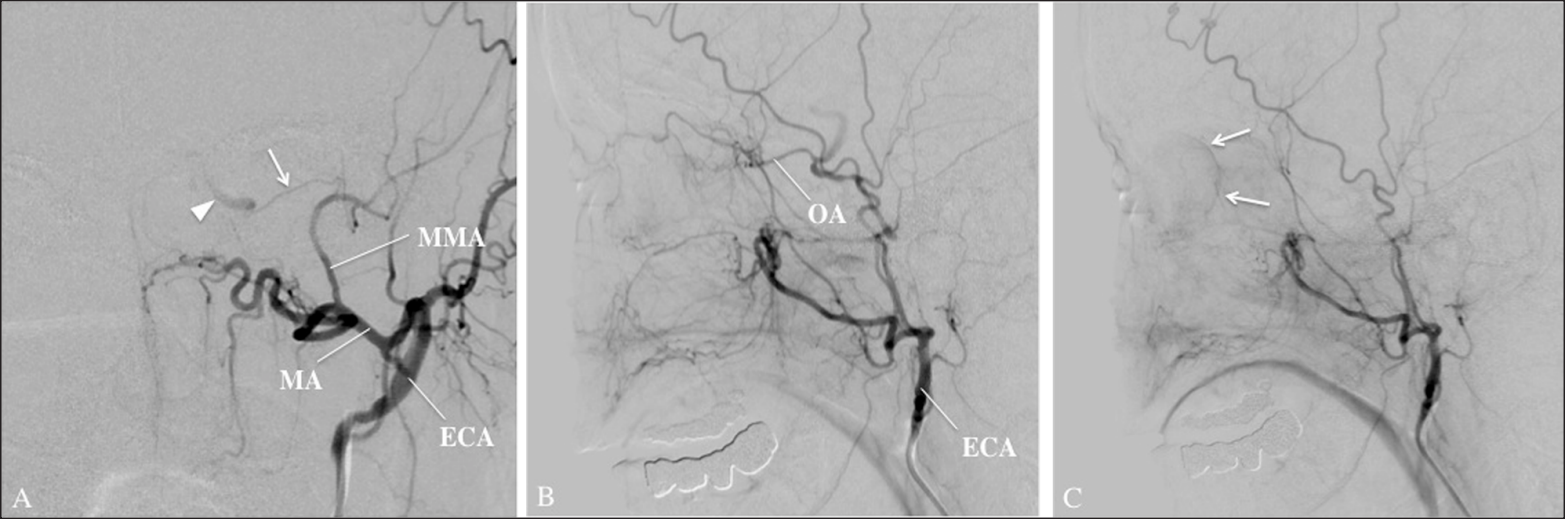
Collateral flow from external to intracranial ICA in a 60-year-old patient with a known stenosis of the left ICA. An antero-posterior view angiography, arterial view, (panel A) after injection of the left ECA shows a small vessel, called the inferolateral trunk (arrow), connecting the petrous part of the internal carotid artery (arrowhead) with the middle meningeal artery (MMA), a major side-branch of the MA. The lateral view angiography, arterial phase, reveals antegrade filling of the OA (panel B) with subsequent visualisation of the retinal blush in the capillary phase (panel C). Embolization in the territory of the ECA in this patient entails high risk of inducing blindness or stroke.

Potentially dangerous anastomoses may exist between branches of the ECA and the eye or the brain. For instance, the MA can have anastomoses with the OA and, via the so-called inferolateral trunk, with the petrocavernous ICA [8] (Fig. 4A–C). Embolization of ECA branches in these patients may lead to inadvertent embolization of ICA branches resulting in unilateral blindness or stroke. This emphasises the importance of a thorough knowledge of the vascular anatomy and of identification of significant anastomoses for a safe endovascular procedure.

## Endovascular embolization of posterior epistaxis

During an embolization, a 4 to 6 Fr guiding catheter is placed in the common carotid artery (from the groin). Through this, a microcatheter will be placed over a microguidewire as close to the bleeding site as possible. The tip of the microcatheter has to be placed in or beyond the distal MA, distal to the origin of the middle meningeal arteries (MMA), to reduce risk of complications. Important extra- to intracranial vascular connections have to be avoided. The goal is to place the tip of the microcatheter in the (proximal) SPA and still have antegrade blood flow in the SPA. Embolization is performed during constant fluoroscopy in order to observe the speed and direction of flow.

One of the most challenging aspects of epistaxis embolization is the stable positioning of the microcatheter at the bleeding site. This depends largely on the condition of the arteries. The elder the patient, the more tortuous the arteries become and the more risk of causing thrombo-emboli or arterial wall dissection that may lead to acute cerebral stroke. In such cases, surgical ligation may be the first treatment option. In patients with bleeding disorders, there is more risk of haemorrhagic complications, not only at the head and neck territory, but also at the groin or iliac arteries.

The moment the microcatheter is at the right location to perform the embolization, the nose packing is removed to observe the bleeding site. Since removal of the packing can result in a new nose bleed, this must only be done when everything is set to embolize the bleeding site. In case of a posterior nosebleed without contrast extravasation on angiography indicating the bleeding site, an embolization can still be performed to prevent recurrence of the nosebleed. The aim of such a preventive embolization is to temporarily devascularize the bleeding site. The aim is to stop the flow in the SPA and reduce the flow in the distal MA. Preventive embolization of the contralateral SPA or the facial artery [9] can be considered, thus blocking potential collaterals to the bleeding side. However, this is only allowed when there is still an active bleeding from a collateral artery. The more arterial collaterals are obstructed, the more risk of ischemia and necrosis (nasal septum, for instance).

The most frequently used embolic agent to embolize a nose bleed are Gelfoam particles that will dissolve in 24 to 48 hours, allowing a revascularization of the temporarily devascularized nose bed. Gelfoam is available in a variety of shapes and sizes including sheets, spherical particles and cubic or pre-shaped forms. Gelfoam can be prepared as torpedoes, slurries or cut pieces. The sizes and shapes are both operator and lesion dependent.

In case of a recurrent nose bleeding despite prior Gelfoam embolization or in case of an active bleeding during the intervention, a more permanent devascularisation is done by microparticle embolization of the bleeding site. Microparticles need to be sized right. If they are too small, they will penetrate to deep and have more risk of obstructing other arteries via collaterals. If they are too large, the devasularization is only proximal and bleeding can still occur through collateral arterial supply. Microparticles of 250–400 µ seem to be the best size. (Micro-) Coils are generally avoided since they only allow proximal embolization and prevent re-intervention in case of recurrent epistaxis. Sometimes, a combination of microparticles and Gelfoam is used. In active bleeding, the embolization is stopped when there is no active contrast extravasation and the supplying and possible collateral arteries are not visualised during angiography controls.

One needs to be aware of the interarterial connections of the nasal supplying arteries and intracranial arteries, mainly the OA. Retrograde flow of microparticles through these connections can cause stroke and blindness (Fig. 4).

Embolization of another bleeding source than the SPA has to be carefully examined, prior to treatment because of the risk of devascularization of important structures. For instance, embolization of side branches of the OA, have a high risk of blindness (Fig. 3). Depending on the bleeding site and aspect of the feeding artery or arteries, other means of embolization can also be used, such as various kinds of glue and/or microcoils in case of (pseudo-) aneurysms or dissections, to control the bleeding.

## Various causes of epistaxis, illustrated with angiographic examples

### Idiopathic epistaxis

**Figure 5:**
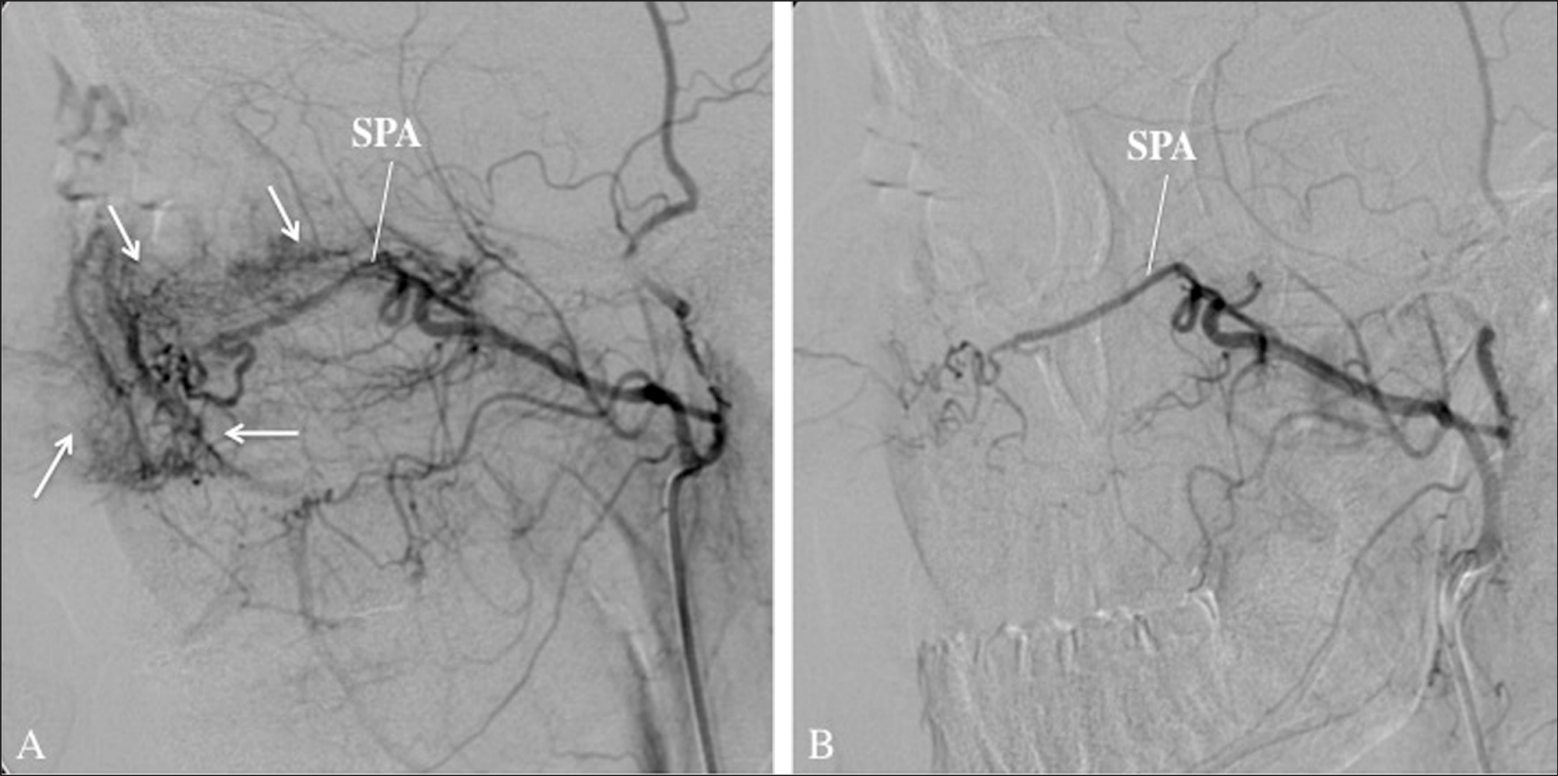
A: Lateral view of a selective angiography of the right MA, arterial phase, shows diffuse hyperaemia of the nasal mucosa (arrows) in the territory of the right SPA. In this patient, the epistaxis was thought to result from diffuse oozing of blood rather than from one large bleeding site. Subsequent embolization was performed by injecting Gelfoam fragments. B: Angiography, lateral view, arterial phase after embolization, shows marked reduction of the hyperaemia. Embolization is usually stopped when the peripheral branches of the SPA do not opacify anymore and the arterial tree seems to be debranched.

About 70% of cases are idiopathic [10] and angiography shows no vascular abnormalities in the majority of the cases. Mucosal hyperaemia can be found in some cases (Fig. 5A–B). In case of recurrent, therapy resistant nosebleeds a preventive embolization of the SPA can be performed, even in the absence of an angiographic proof of epistaxis [11].

### Post-traumatic epistaxis

Epistaxis can occur after maxillofacial trauma. In case of massive exsanguination and hypovolemic shock the patient should be referred for immediate surgery, often sacrificing large external carotid artery branches. In a relatively haemodynamic stable patient, a selective endovascular embolization of the site of the bleeding can be attempted, to save normal arteries. If injury to the ethmoid arteries is responsible, endovascular intervention is generally not an option and the patient should be referred for surgical ligation.

### Post-operative epistaxis

**Figure 6:**
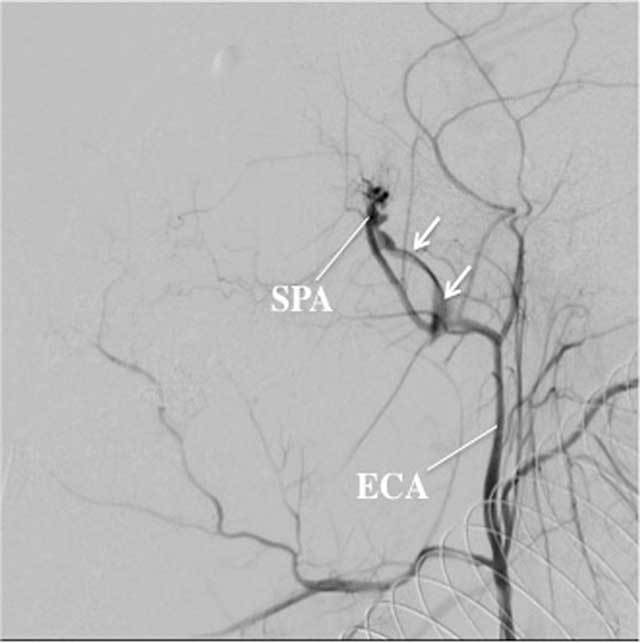
Angiography, lateral view, arterial phase, of the left ECA in a 19-year-old man with uncontrollable epistaxis less than 24h after surgical removal of a nasopharyngeal mass. At the level of the SPA, a curvilinear contrast extravasation can be seen along the posterior margin of the nasopharynx, mimicking the appearance of a vein, known as the *pseudo vein sign*. Endovascular embolization with coils and Gelfoam fragments successfully stopped the bleeding.

**Figure 7:**
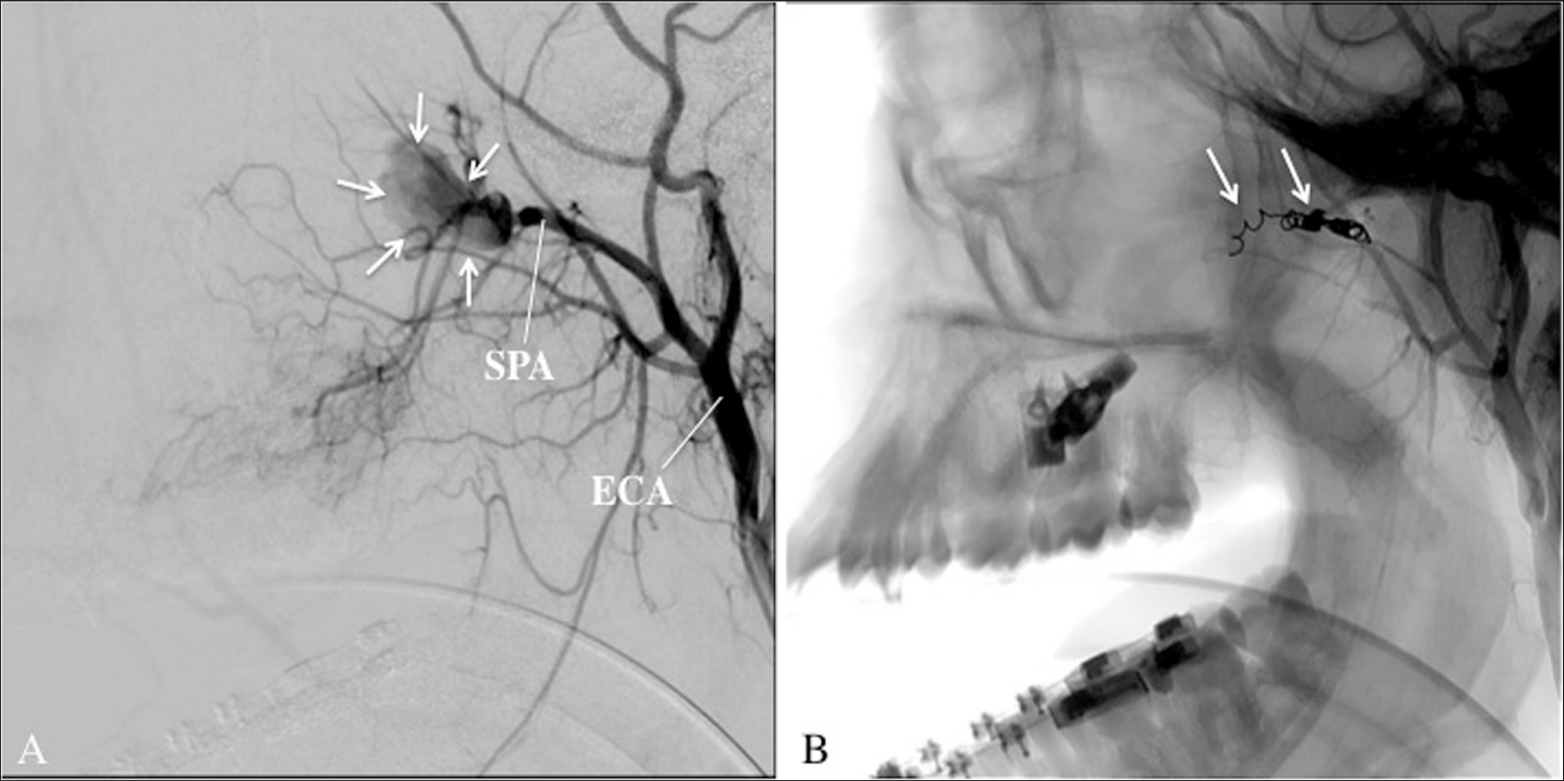
A: Angiography, lateral view, arterial phase, of the left ECA in a 26-year-old man presenting with massive epistaxis 3 weeks after orthognatic surgery. A large pseudo aneurysm (arrows) of the left SPA can be clearly depicted. B: This patient was successfully treated with endovascular coiling (arrows) at the level of the arterial wall defect. Coils were placed at the level of the defect, as well as proximal and distal of the defect in order to control the bleeding site in both an antegrade and retrograde way.

Epistaxis after surgery in and around the nasal cavity is a well-known complication, that can be potentially life threatening. Angiographically, an active bleeding can be identified by visualisation of contrast extravasation (Fig. 6). Sometimes a delayed epistaxis occurs due to development of a pseudo aneurysm [12] (Fig. 7A–B). In case of large arterial wall defects the use of coils may be necessary to stop the bleeding.

### Systemic vascular disorders

**Figure 8:**
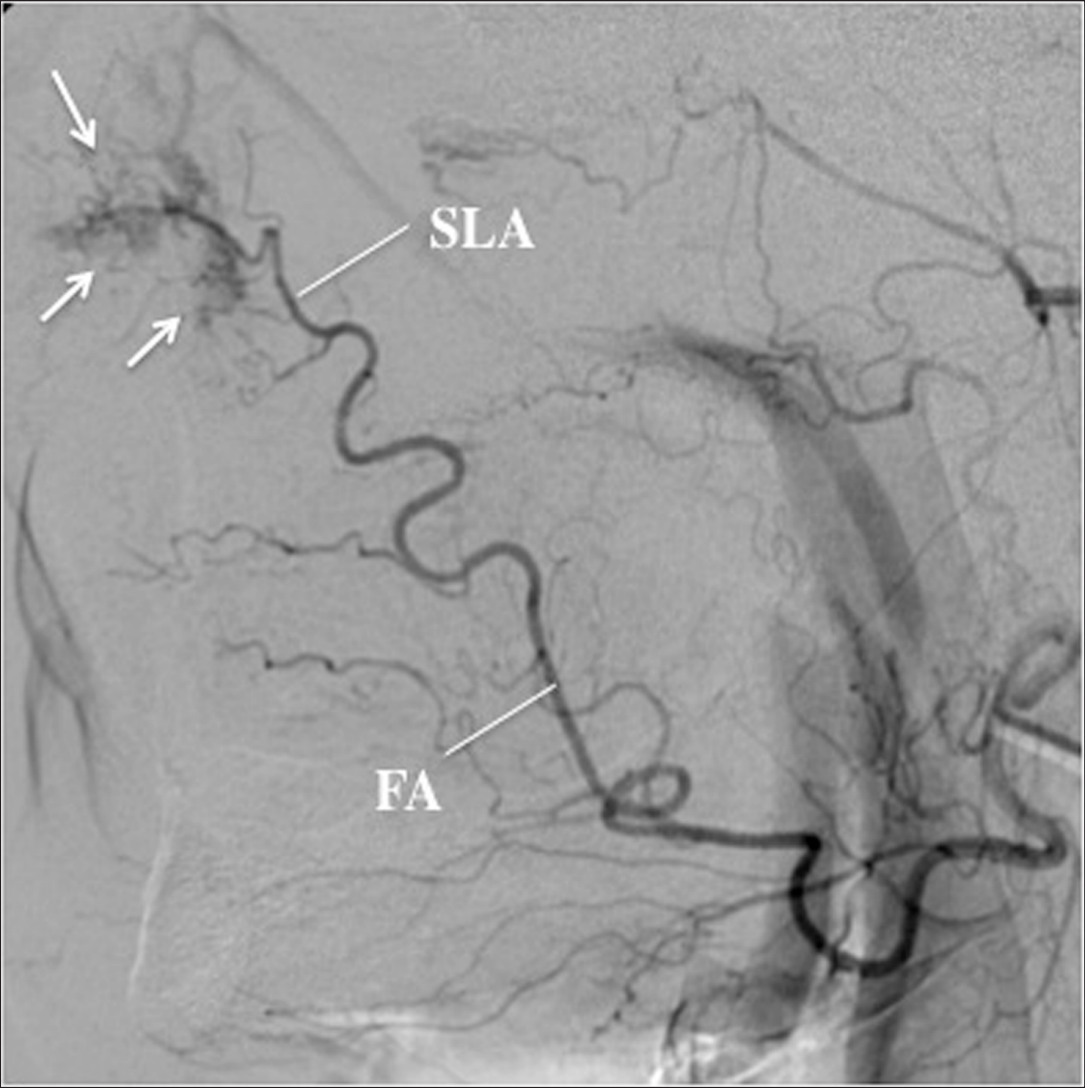
Selective angiography, lateral view, of the left FA in a 62-year-old Rendu-Osler-Weber patient presenting with mild but therapy resistant epistaxis. Because of recurrent epistaxis in the past, several endovascular interventions at the level of both SPA’s were already performed. Pathological vessels in the SLA territory, anterior in the nasal cavity, were held responsible for the current episode. The feeding arteries were embolized using microparticles and glue.

Haemorrhage due to vascular malformations can occur anywhere in the body, including the nasal cavity. Rendu-Osler-Weber disease or hereditary haemorrhagic teleangiectasia (HHT) is a systemic vascular disorder affecting mucosa, the skin, the lungs, the gastrointestinal tract and the central nervous system. Typically, it causes recurrent episodes of epistaxis originating from spontaneous bleedings from teleangiectasias of the nasal mucosa [13] (Fig. 8). In contrast to the idiopathic group, angiographic abnormalities can be seen in nearly all patients [14]. Treatment of epistaxis in these patients is generally very frustrating, with high rate of recurrence. The main goal is to reduce the number and severity of nosebleeds. Because re-intervention is often required, the use of coils is avoided. Long-term improvement is rarely achieved [15].

### Tumor-induced epistaxis

**Figure 9:**
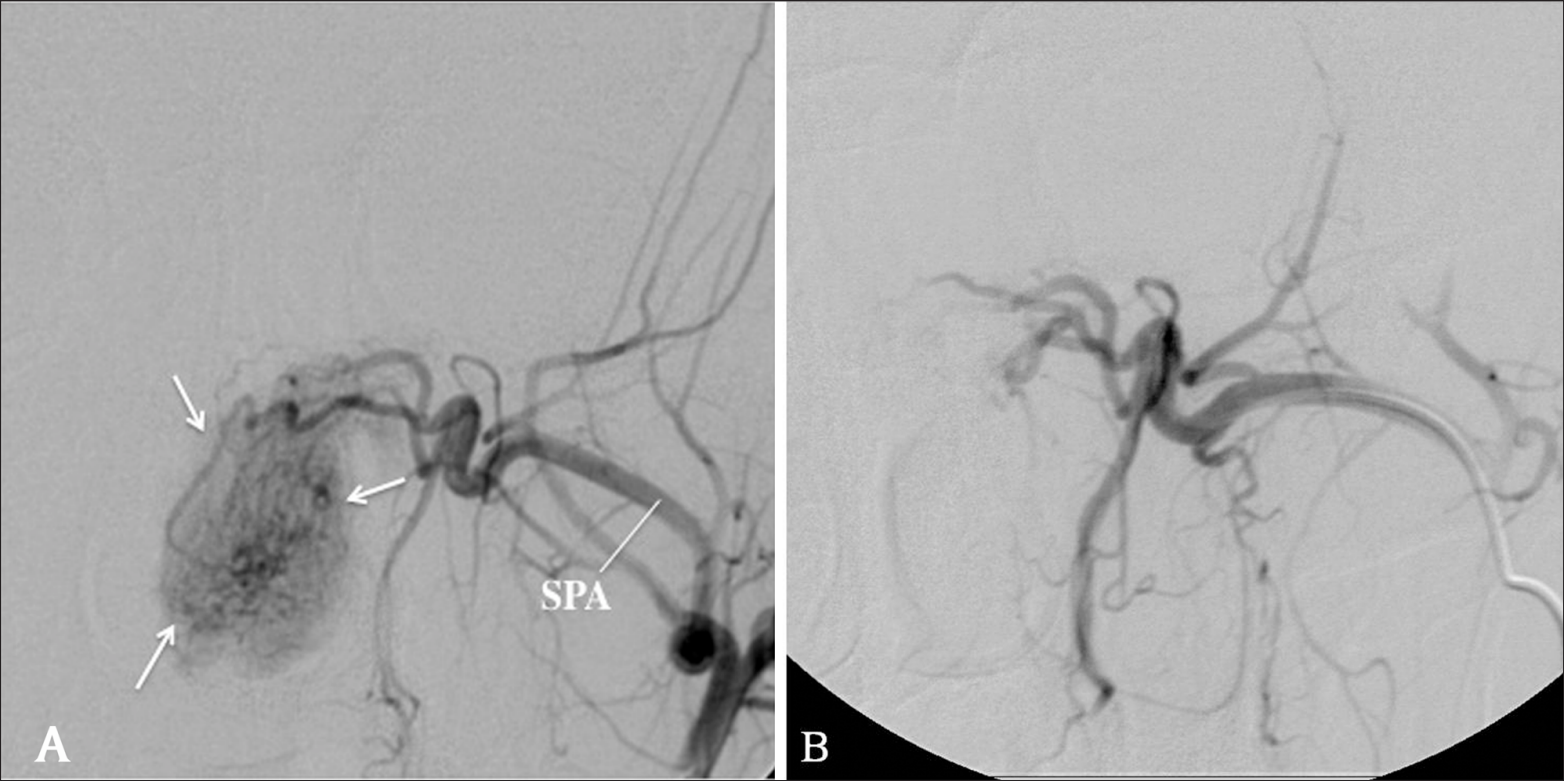
A: Selective angiography of the left MA, antero-posterior view, arterial phase, in a 19-year-old man diagnosed with a juvenile angiofibroma, shows a well-circumscribed tumor blush (arrows), vascularized by the SPA. No other arteries were found to vascularize the tumor. B: Superselective angiography, antero-posterior view, arterial phase, with a microcatheter in the SPA shows the devasularized tumor after embolization with microparticles.

Almost any sinonasal tumor can cause epistaxis. The list of sinonasal tumors that can cause epistaxis is long, including nasopharyngeal carcinoma, hemangioma, hemangiopericytoma and metastatic disease. One specific type of nasopharyngeal tumor, juvenile nasopharyngeal angiofibroma, accounts for the majority of tumor-related epistaxis episodes. It is a benign tumor originating from the posterolateral wall of the nasal cavity, occurring almost exclusively in young males. Embolization of the main feeding arteries can be performed to stop epistaxis and as a preoperative treatment in reducing significant blood loss during surgery [16,17,18] (Fig. 9A–B). Angiography might be very useful in pre-operative planning, since some angiofibromas are vascularized by both the ICA and ECA with rich collateral arterial supply bilaterally [19,20].

## Conclusion

Epistaxis is a common medical condition. The majority of epistaxis episodes are found anterior in the nasal cavity and are self-limiting. However, posterior epistaxis is more challenging to treat. In case of persistent or recurrent bleeding despite proper conservative measures, including anterior and posterior nasal packing, transarterial embolization is a valid treatment option. The success and complication rate is comparable to surgery. However, serious complications like blindness and stroke are more often associated with embolization. On the other hand, an endovascular treatment approach is much less invasive, cheaper, and usually does not require general anaesthesia. Therefore, in daily practice, the choice of treatment should be taken on an individual patient-based evaluation, taking into account present comorbidities, the location of the bleeding, available equipment and individual vascular anatomy.

## Competing Interests

The authors declare that they have no competing interests.
